# Mr. Maurice, on the Coincidence of Diseases

**Published:** 1803-01-01

**Authors:** J. Maurice

**Affiliations:** Marlborough


### *To the Editors of the Medical and Physical Journal.*

GENTLEMEN,

AS it has been a prevailing opinion among medical men, that no two diseases could exist and go through their different stages, in the same person, at one time, I am induced to transmit you the following cases to be inserted in your valuable Journal, if you think them sufficiently interesting. I am, &c.

*Marlborough, Dec.* 8, 1802. J. MAURICE.

---

I was requested to inoculate a child in this town with vaccine virus on the 16th of September, at which time the measles were very prevalent here and in the neighbourhood. On the third day after inoculation, this child was attacked with the usual symptoms of measles, the eruptions appearing on the sixth day after vaccination. I particularly attended to the state of the arm, but could not discover the smallest variation from its regular progress, neither were the symptoms of rubeola in the least lessened or retarded. On the 24th, another of the same family was vaccinated, which was also seized with the measles; both diseases went on regularly as in the former case, only that this child suffered much from a pneumonic affection, which can only be attributed to that disposition, which measles in in general have to produce such complaints. I have not been so particular as to state the daily progress of the diseases, because it could not tend to prove any thing more than I have already advanced.

Wishing to ascertain what effects might be produced by inoculating others, or whether one or both diseases may be communicated, I accordingly took MATTER* from the arms of these children, and inoculated another family, consisting of the mother and five children; the children went through the usual stages of vacciola, and without one symptom of rubeola.† But the mother did not receive the infection, notwithstanding several attempts were made. She informs me, that when an infant, her mother caught the small-pox, of which she died; but no one ever discovered that she (the infant) had a sympton of the disease.

In the course of my practice, I have met with two or three cases similar to this; where I could not communicate the vaccine disease, although repeated attempts were made with the most recent virus. I do not know how to account for this phenomenon‡; as it has been clearly proved by the Rev. G. C. JENNER and others, that persons who have had the small-pox may be infected with the cow-pox.

As much has been already said by so many able practitioners, respecting the superior advantages the vacciola possesses over the variola, the following will afford nothing new, but only tends to corroborate it still further, for which reason I lay it before you.

On the 26th of October, the small-pox made its appearance on a young man of the parish of Chissleden, who caught the contagion in London. The alarm was consequently great, and it was determined to inoculate all those who were supposed to be in danger, many of whom were relatives of this poor man, and lived under the same roof.

The medical gentleman, who attended this man, promised to inoculate them immediately, which he neglected doing,

* The matter was taken on the 9th day of vaccination, when the morbillous eruptions were at their height.

† May not this be sufficient to prove that every virus is sui generis, and that the vulgar notion of scrofula being communicated by small-pox inoculation is highly improbable; in my opinion, it is only excited into action by the small-pox.

‡ Except that the want of susceptibility in some constitutions to receive either the variolous or vaccine diseases may be considered as the cause.

doing. On the 30th they made application to me, when I vaccinated fourteen in number, some of whom, wishing to give it a fair chance, (as they termed it) lived in the same house with the variolous patient, two days previous to his death, where they still reside. The poor man died the 4th of November, being the ninth day after the eruptions became visible. Every one of these patients went through the vaccine disease without any interruption, and it is to be remarked, that although several of them were exposed to a variolous effluvia, yet no pustules appeared.

Out of nearly two hundred patients that I have vaccinated, not one of them has suffered materially from inflammation of the arm.

P. S. Since the above was written, several poor people of the same parish have inoculated themselves with vaccine virus, by means of a razor, making a long and deep incision; consequently, they have very bad arms, which is owing probably to the improper mode of inserting it; for, Dr. JENNER asserts, " that when the adipose membrane is wounded, a violent disease is produced," or, from the matter being taken at an improper period. These circumstances, I fear, may be the means of bringing the vaccine disease into disrepute.

